# Anti-Suicidal Efficacy of Repetitive Transcranial Magnetic Stimulation in Depressive Patients: A Retrospective Analysis of a Large Sample

**DOI:** 10.3389/fpsyt.2019.00929

**Published:** 2020-01-08

**Authors:** Mohamed A. Abdelnaim, Berthold Langguth, Markus Deppe, Alexey Mohonko, Peter M. Kreuzer, Timm B. Poeppl, Tobias Hebel, Martin Schecklmann

**Affiliations:** ^1^Department of Psychiatry and Psychotherapy, University of Regensburg, Regensburg, Germany; ^2^Department of Psychiatry, Psychotherapy, and Psychosomatics, Medical Faculty, RWTH Aachen University, Aachen, Germany

**Keywords:** transcranial magnetic stimulation, suicide, suicidal ideation, depression, brain stimulation, rTMS

## Abstract

**Background:** Suicide is a major public health problem. About 90% of suicide victims have one or more major psychiatric disorder, with a reported 20-fold increased risk for suicide in patients with affective disorders in comparison with healthy subjects. Repetitive transcranial magnetic stimulation (rTMS) has been established as an effective alternative or adjunctive treatment option for patients with depressive disorders, but little is known about its effects on suicide risk.

**Objective:** For the assessment of the effectiveness of rTMS on suicidal ideation and behaviors, we performed a retrospective analysis of a large sample of patients with depressive disorders, who were treated with rTMS.

**Methods:** We analyzed the records of 711 TMS in- and out-patients with depressive affective disorders in a tertiary referral hospital between 2002 and 2017. Out of these patients we were able to collect Hamilton depression rating scale (HAMD) data of 332 patients (180 females, 152 males; age range 20 to 79 years; mean age 47.3 ± 12.3) for which we analyzed the change of suicidal ideation by using item 3 (suicidality) of HAMD.

**Results:** Out of all 711 patients treated with rTMS for their depression, one patient (0.1%) committed suicide during the TMS treatment. In the statistical analysis of the subsample with 332 patients there was an overall amelioration of depressive symptoms accompanied by a significant decrease in the suicidality item with a medium effect size. Decrease in suicidality was not inferior to changes in other items as indicated by effect sizes. Forty-seven percent of patients showed an amelioration in suicidality, 41.3% of patients did not show a change in their suicidality’s scores, and 11.7% of patients showed an increase in suicidality’s scores from baseline to final rating. Correlation of item 3 (suicidality) and item 7 (drive) demonstrated a significant positive association, revealing improved drive with a parallel decreased suicidality.

**Conclusion:** Based on the proposed data, there is no evidence that rTMS increases the risk for suicide during the course of the treatment. Conversely, rTMS tends to reduce suicidal ideation. Our findings call for further rTMS controlled studies using large sample sizes and specific suicidality assessment measures to obtain more conclusive results.

## Introduction

Suicide is a major public health problem. World-wide nearly 1 million lives are lost each year because of suicide and 3–5% adults committed at least one suicide attempt at some point in their life (1–3). Although those numbers of people dying of suicide are already high, according to the World Health Organization they are presumably underestimated, and that for each adult who dies by suicide, there may have been more than 20 others attempting suicide (4). Suicidal behavior is a very complex, multi-factorial behavior, involving several medical-biologic, psychosocial, and cultural components (5). The coexistence of mental disorders in suicidal subjects has been widely studied. Psychological autopsy studies from different parts of the world found high prevalence of mental disorders in suicide victims, indicating that around 90% of them have one or more axis I (mostly untreated) major psychiatric disorders at the time of their death (6–8). Among those disorders, mood disorders represent the major risk factor for suicidal ideation and suicide attempts (9, 10, 11). In comparison with healthy subjects, a 20-fold increased risk was reported for the patients with affective disorders (12).

Moreover, affective disorders do not only increase the risk of suicidal ideation and tendency to attempt suicide but also the risk of a suicidal death. It is estimated that between 50 and 70% of all suicide victims are related to depressive and other mood disorders (ICD-10 F3); (13–15), with increased lethality of suicide attempts in patients with major affective disorders being reported (16).

The general recommendation to reduce suicide risk is the effective treatment of the underlying depressive disorder, with medications and psychosocial interventions including psychotherapy being the main therapy modalities (17). However, there exist several difficulties. First, neither antidepressants nor psychotherapy work fast enough for reduction of suicidal ideation. Second, even for medications with well-established reduction in suicidal behavior such as lithium, effectiveness is limited (18). Third, the prescription of antidepressants may offer the depressed patient a potential suicide method, e.g., by intoxication with tricyclic antidepressants, which are known for their cardiotoxicity (19). Fourth, the reporting of an association between increased suicidal ideation and antidepressant treatment in controlled clinical trials of antidepressants has added more difficulty to the connection between depression, its pharmacological treatment, and suicidality (20–23). The mechanism through which antidepressant medication might increase suicidality is yet not understood. A possible explanation for conflicting results concerning the relationship of antidepressant treatment and suicide risk is a transient increase of suicidal behavior after initiation of treatment. In detail, it has been suggested that antidepressants may energize depressed patients before they lead to mood improvement (24). This period of increased impulsivity with ongoing desperation may be related to increased suicidal behavior. Also, side-effects of antidepressants such as worsening of irritability, agitation, and insomnia could ease suicidal ideation and behavior (25, 26). Other results suggested that suicidal behavior in patients taking antidepressants is mostly the consequence of the lack of antidepressant effect and is rarely the result of suicide-inducing potential of antidepressants (27). All of the above-mentioned concerns indicate the need for more safer and more effective interventions to reduce suicide risk.

Beside pharmacotherapy and psychotherapy brain stimulation methods have gained increasing relevance in the treatment of depression during the last decades (28). Electric convulsive therapy (ECT) is an established antidepressant method with clearly proven efficacy for fast improvement of suicidal behavior, however, the use of ECT is limited by safety concerns and adverse effects (29).

Repetitive transcranial magnetic stimulation (rTMS) is a non-invasive brain stimulation technique, that has emerged in the last 30 years and is applied in various neuropsychiatric conditions (30). rTMS uses pulsed magnetic fields with an intensity of up to 3T to induce neuronal depolarization in superficial cortical areas. For the treatment of depressions several stimulation protocols have been developed with high frequency rTMS of the left dorsolateral prefrontal cortex being the most established protocol (30).

In 2008, rTMS was approved for use by the FDA as a treatment for major depression for patients who do not respond to at least one antidepressant medication in the current episode (31) (FDA approval K061053).

The efficacy of rTMS is supported by multiple double blind, randomized controlled trials (32–34) that are summarized in several systematic meta-analysis, reviews, and evidence-based guidelines (30, 35). In general, the focus was more set on the efficacy of rTMS in patients with TRD (treatment resistant depression), considering its anti-depressive properties to be “obvious, opening interesting prospects, in particular in the treatment of pharmaco-resistant major depressive patients” (36). However, the effectiveness of rTMS has not only demonstrated in treatment refractory patients. rTMS has also been investigated in less treatment-resistant patients who might benefit even more from TMS than patients with pharmaco-resistant depression (37).

Due to its good tolerability and because it is free of the side effects that commonly accompany antidepressant medications, it would also seem attractive to patients, who are not willing to take a medication (38).

Regarding safety and adverse events of rTMS, side effects like local pain, discomfort, headache, vasovagal syncope are reported, and the induction of an epileptic seizure is the most important safety concern of rTMS treatment (39). However, the incidence of seizures with TMS is relatively low and is less evident than that with current antidepressant medications (40).

In comparison to ECT, although the latter seem to have higher response rates in treatment-resistant depression (41), rTMS does not require anesthesia or muscle relaxation (does not require induction of a seizure). Moreover, the magnetic stimulation is delivered in a focal manner at a chosen cortical target rather than affecting the entire brain as with ECT (42), which significantly reduces side effects (43). Furthermore, there is no evidence of cognitive impairment connected to TMS, also most adverse events of TMS are mild to moderate in intensity, which leads to a low discontinuation rate due to adverse events during the treatment course (40).

These summarized data with respect to efficacy and side effects propose rTMS as promising alternative or additional intervention in the treatment of depression. Some researchers have already reported a decrease of suicidal ideations following treatment with rTMS (33, 44–46). These results however were considered as preliminary because most of them were not sham-controlled with mostly very limited suicide assessment measures and relatively small sample sizes (47). In their study of open-label accelerated TMS (aTMS) over 2 days in 14 depressed patients, Holtzheimer et al. reported that only one patient showed increased suicidal ideation (48).

In a Chinese randomized controlled trial, it has been found that rTMS combined with medications reduces suicidal ideation in elderly patients with depression (49). In their study of applying high frequency TMS to the left dorsolateral prefrontal cortex (DLPFC) on 19 adolescents with treatment-resistant depression, Croarkin et al. reported improvement in suicidal ideation across 30 sessions associated with improvement in depressive symptom severity (50).

Our goal from this study was to investigate the relationship between rTMS and suicidal risk in a more naturalistic sample, which avoids the disadvantage that patients with high risk for suicidality were typically excluded from rTMS treatment studies. We conducted a retrospective analysis of a large dataset from patients that received rTMS for the treatment of depression in a real-world clinical setting of a tertiary referral hospital.

## Methods

The analyses and publication of this retrospective work was approved by the ethic committee of the University of Regensburg (ethic vote: 16-104-0223). We revised and analyzed the records of 711 patients with mood disorders, who were treated with rTMS in the Center for Neuromodulation Regensburg (Germany) between 2002 and 2017 excluding patients receiving sham. One twenty-eight out of them were treated more than one time with rTMS. The patients are referred to us either from our different inpatients’ wards or from our outpatient clinic *via* the responsible psychiatrist. Those are typically the patients who failed to show an adequate response to standard therapies. Suicidality is not an exclusion criterion *per se* except for acute suicidality which would be associated with hospitalization in a closed station.

For this sample of 711 patients we report the number of committed suicides within the course of the treatment with rTMS.

In addition, we analyzed a subsample with respect to the effect of rTMS on suicidal ideation based on the Hamilton depression rating scale (HAMD). The inclusion criteria for the retrospective analysis were: rTMS-naïve (only the patient’s first treatment with rTMS was considered), primary diagnosis of a depressive disorder [including bipolar disorder currently depressive episode (ICD-10: F31), major depressive disorder (ICD-10: F32), and recurrent depressive disorder (ICD-10: F33)], a complete documented HAMD at beginning and at the end of rTMS treatment, absence of a serious somatic illness. Both in- and outpatients were included.

We were able to collect data of 332 patients, who met the above-mentioned criteria including 180 (54.2%) females and 152 (45.8%) males with an age range between 20 and 79 years (47.3 ± 12.3). Patients were treated with a minimum of 6 up to 50 sessions (17.0 ± 6.5). Patients received different rTMS protocols, most of them received 20 Hz stimulation on the left prefrontal cortex (**Table 1**).

**Table 1:** Frequency of repetitive transcranial magnetic stimulation protocols performed.

Protocol (frequency—site—daily pulses)	Absolute number of patients	Relative number of patients
20 Hz—left prefrontal—2,000	261	78.6%
10 Hz—left prefrontal—2,000	15	4.5%
10 Hz—left prefrontal—1,000	10	3.0%
20 Hz—right prefrontal—2,000	3	0.9%
1 Hz—right prefrontal—1,000	1	0.3%
10 Hz—anterior cingulate cortex—2,000	12	3.6%
20 Hz—left prefrontal followed by 1 Hz—right prefrontal—2,000	11	3.3%
iTBS—left prefrontal followed by cTBS—right prefrontal—2,400	19	5.7%

The analysis was based on the scores of the 21-item Hamilton Depression Rating scale (HDRS or HAMD) (51), which is considered to be a reliable depression scale regarding internal consistency, inter-rater and test-retest reliability (52). We focused on the changes in the item 3 (suicidality). This item evaluates the presence and severity of suicidal thoughts/action on a 4-point scale, leveling from absence of suicidal thoughts (score 0) as the lowest score, the feeling that life is not worth living (score 1), presence of death wishes (score 2), suicide ideas (score 3), till suicidal attempts (score 4), as the highest score.

We compared the baseline scores (at beginning of treatment) with those at end of treatment, trying to find out if the scores would increase or decrease after the rTMS treatment. This was done for all single items and also the sum score of the HAMD to investigate whether change in suicidality went in a similar direction as change of other depressive symptoms. For item 3 we also looked in the change of the frequency of the single grades of the question.

We’ve also tried to correlate the results with the scores of item 7 (work and abilities), which focuses mainly on the patient’s energy and drive. The motivation for this analysis was twofold. Firstly, it is assumed that energizing a depressed patient through medication can also increase the risk of suicidality. Secondly, we aimed to investigate whether improvement/worsening the suicidal ideation is attributable to parallel improvement/worsening of patient’s energy level. To see if a putative association is specific we did this correlation for all items and also for the sum score.

All data were analyzed using Statistical Package for the Social Sciences (SPSS; IBM SPSS Statistics for Windows, Version 24.0.0.1). The significance level was set at *p* < 0.05 for all analyses. Significant findings which survived correction for multiple comparisons according to Bonferroni were separately marked. For pre-post contrasts we used Wilcoxon rank sum tests. For cross-tab analysis we used the chi-squared test of independence. For correlations we used Spearman correlation coefficients. Effect sizes were reported by Cohen’s d (53), and were calculated according to Lenhard & Lenhard (54).

## Results

From the above-mentioned 711 patients, only one patient (0.1%) committed suicide during the TMS treatment. In this single case the role of TMS remains unclear. Nine years prior to his suicide, this patient had already received a successful 15-days rTMS treatment course. Re-hospitalization occurred because of another depressive episode. After a short period, the patient was discharged from the hospital. Due to re-emerging of suicidal impulses immediately after hospital discharge the patient was hospitalized again. After a month of hospitalization, because of failed response to antidepressants, and also the prior treatment success of TMS, a new treatment course was proposed. After the first TMS session, he was seen by a doctor. He appeared to be calm, claimed to be in a good mood and denied having any suicidal thoughts. Also, in his conversations with nursing staff and a phone call with a relative afterwards, the patient appeared to be in a stable condition free from suicidal ideation. Later on that day, he left the clinic ward, didn’t answer his cell phone anymore and sadly then committed suicide. Finally, the reasons for the suicide remained unclear. To which extent the underlying depressive disorder, the used therapies (medication, psychotherapy, rTMS), or any other factors contributed to the suicide, couldn’t be identified.

**Figure 1:**
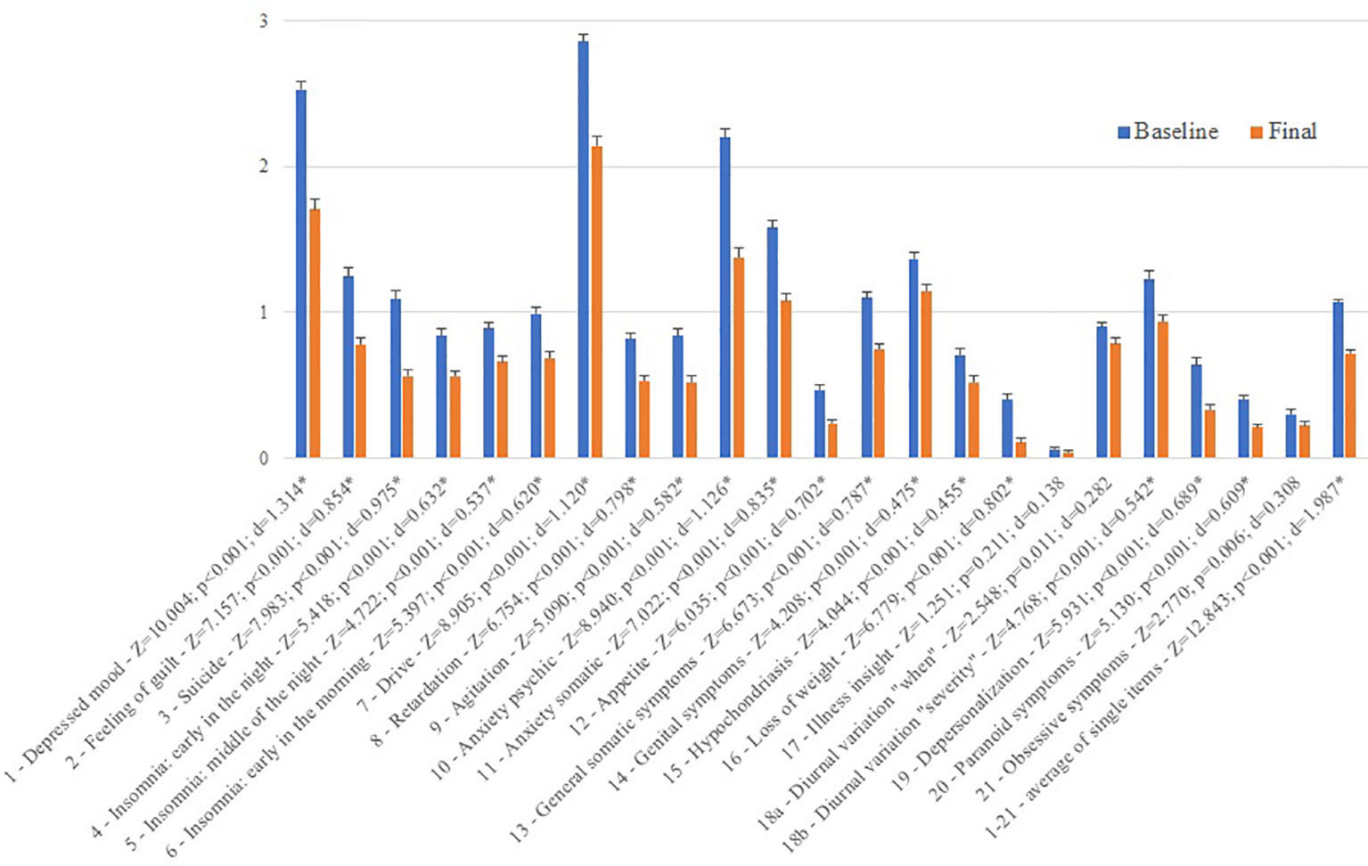
Difference between baseline and final scores of the Hamilton Depression Rating Scale items. *indicate significant Wilcoxon tests which survived correction for multiple comparisons. Z corresponds to the z-value of the test statistic and d to the effect size. Please note that for reasons of similar scale the overall score is shown not as sum but as average score.

In the statistical analysis of the subsample with 332 patients there was an overall amelioration of depressive symptoms accompanied by a significant decrease in the suicidality item with a large effect size. Only three items did not survive correction for multiple comparisons. Decrease in suicidality was not inferior to changes in other items as indicated by effect sizes (**Figure 1**). The chi-square test of independence was significant (χ² = 43.318; df = 12; p < 0.001) showing an association of the severity of depression before and after rTMS.

The number of patients who scored 0 increased from 118 at the baseline rating, to 208 patients at the final rating. Also, less patients scored 1, 2, and 3 at the final rating (81, 33, 14 respectively) compared to the baseline rating (111, 72, 34 respectively). No patient scored 4 at the final rating compared to three patients at the baseline rating.

**Figure 2:**
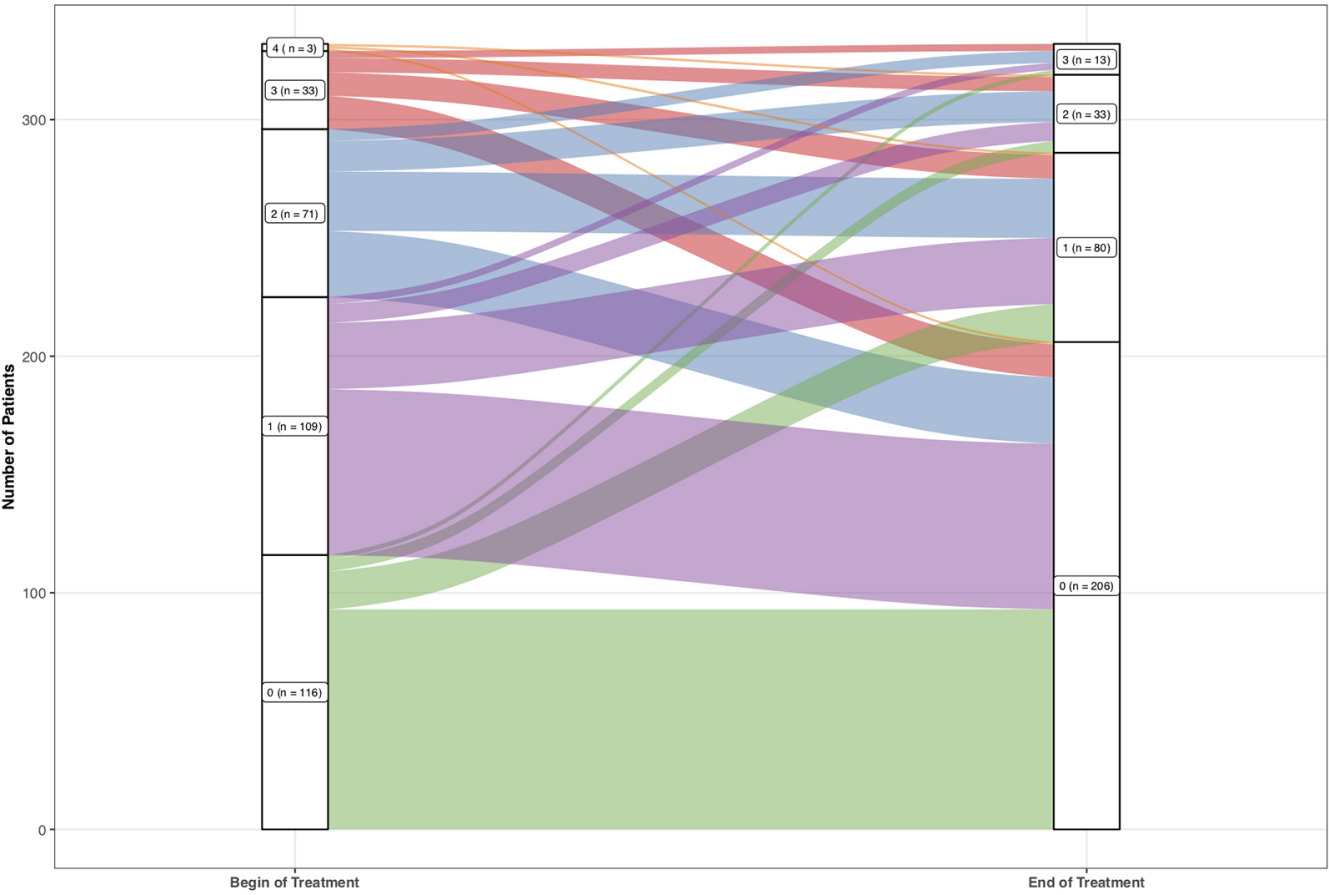
Absolute frequencies of the single values of the suicidality item 3 of the Hamilton Depression Rating Scale.

One hundred fifty-six (47.0%) patients showed an amelioration in suicidality. One patient showed amelioration of 4 points (0.3%), 15 of 3 points (4.5%), 39 of 2 points (11.8%), and 101 of 1 point (30.4%). One hundred thirty-seven (41.3%) patients did not show a change in their suicidality’s scores. Thirty-nine out of the 332 (11.7%) patients showed an increase in suicidality from baseline to final rating as indicated by increase from 0 during baseline to 1, 2, or 3 during the final rating or from 1 during baseline to 2 or 3 during final rating or from 2 during baseline to 3 during final rating. Twenty-nine patients showed worsening of the scoring of 1 point (8.7%), 8 of 2 points (2.4%), and 2 of 3 points (0.6%). No patient increased to the maximum score of 4. For absolute numbers see (**Figure 2**).

Non-parametric Spearman correlation of item 3 (suicidality) and 7 (drive) demonstrate a significant positive association (r = 0.196; p < 0.001), that means that patients showed improvement of their suicidality alongside with increased drive. Items 1 (r = 0.263; p < 0.001) and 2 (r = 0.265; p < 0.001) and sum score (r = 0.426; p < 0.001) also correlated significantly on a Bonferroni corrected level, the other items not.

## Discussion

Suicidal ideation is a severe symptom of depressive disorders and a huge concern for mental health providers. Only few treatments for suicidal ideation and behavior (e.g., lithium, ECT) are available and these are only partially effective. There is an enormous need for more convenient interventions.

Therefore there is a great need to firstly develop new treatment modalities with a rapid effect on suicide risk in order to prevent suicide (18, 47) and to secondly assess the effects of established antidepressant treatments on suicidal ideation.

Transcranial magnetic stimulation represents a non-invasive, feasible, effective, and safe treatment option for depression (55). There are no published reports that TMS increases suicide risk (56). However, there is only relatively few systematic research about the effect of rTMS on suicidality. In our analysis, which comprised a large sample of 711 patients, we found an overall improvement of suicidal ideation represented in item 3 of the HAMD score. The improvement in this item corresponded well to the overall improvement in the total HAMD score; 88.3% of all patients showed either improvement or stability of their suicidal ideation score, compared to 11.7% in which the score worsened under the TMS treatment course. Together with the significant chi-square test, we can conclude that we found no evidence that rTMS systematically increases suicidality.

Also, we found a positive correlation between improved patient’s drive (indicated by item-7 score) and improved suicidality (indicated by item-3 score). Changes in suicidality were also significantly associated with change in the total score and with change in the items 1 and 2 (depressed mood and feeling of guilt). The changes of all other items showed smaller and non-significant correlations. We interpret this in a way that change in suicidality is associated with changes in the main symptoms of depression considering that suicidality is also one main symptom of depression. Thus, we found no hint that rTMS induces an energizing effect without improvement of mood and that such a constellation may increase suicidality.

Our results get in line with some prior research revealing alleviation of suicidal ideation under TMS treatment. Hadley et al. reported in their trial on the effect of rTMS in patients with treatment-resistant unipolar or bipolar depression improvement of all measured dimensions, and most importantly, that suicidal ideation diminished in 67% of the patients already after the first week of treatment (57). In another 3 days-trial of delivering high doses of rTMS on left prefrontal cortex in patients with suicidal ideations, a more rapid decline in suicidal thinking has been observed in the active rTMS group compared to the sham group. The rather intense stimulation schedule (delivering 54,000 stimuli over 3 days), was feasible and safe with minimum side effects, and particularly with no worsening of suicidal thinking (58). Desmyter et al. examined in a randomized, sham-controlled trial, the effects and safety of accelerated intermittent Theta Burst Stimulation (iTBS) on suicide risk by using the Beck scale of suicide (BSI). A significant decrease of the BSI score over time was observed for both active and sham stimulation and unrelated to depression-response. There was no worsening of suicidal ideation, and the decrease in suicide risk lasted up to 1 month after baseline, even in depression non-responders (46). Weissmann et al. compared the effect of bilateral, left unilateral DLPFC, and sham rTMS on suicidal ideation in patients with TRD (who failed to respond to at least two antidepressant medications). They found a significant superiority of bilateral rTMS to sham rTMS in reducing suicidal ideation, with only a small portion of this reduction in suicidal ideation attributable to the improvement of overall depressive symptoms (59).

In our sample, among 711 patients that were treated with rTMS there was only one single suicide. For this suicide no obvious causal relation with rTMS treatment could be established. In most of the 711 patients, rTMS was not performed in the context of a clinical trial (where a high suicide risk is frequently an exclusion criterion), but as compassionate use treatment because of treatment resistance to medication and psychotherapy. This means that the sample consisted of treatment resistant patients with a rather high suicide risk. Our findings not only give a powerful indicator for the safety of TMS regarding suicidality, but also present TMS as a potential therapy option to reduce suicidal ideation.

This notion is supported by the presumed mechanism of action of prefrontal TMS namely the improvement of cortical-limbic regulatory control over emotional drive (60). The suicidal crisis has been described as a dysfunctional brain event, related to changes in the prefrontal cortex which leads to deficient regulation of the emotional state (61). Baeken et al. investigated how accelerated iTBS (aiTBS) may influence brain perfusion and suicidal thoughts using arterial spin labeling (ASL) fMRI, they found that both active and sham aiTBS resulted in prompt decreases in suicidal ideation, but specifically sham aiTBS has significantly attenuated frontopolar perfusion in relation to reductions in BSI scores. They interpreted those findings that in accelerated neurostimulation paradigms, placebo responses are related to perfusion decreases in brain areas associated with higher cognitive processes such as the default mode network, resulting in suicidal ideation attenuation (62).

Whereas our results demonstrate the effectivity of rTMS on suicidal ideation, we are aware of several limitations of the study. Firstly, this is a retrospective analysis, secondly rTMS was applied as an add-on treatment. Thirdly there was no sham control and fourthly there were no follow-up measures. Additionally, the study depended on a single item of the HAMD scale and not a specific assessment scale for suicidality e.g., the Beck Scale for Suicidal Ideation (63). Also, only three subjects of the sample had endorsed suicidal behavior (scoring 4 at the item 3 at baseline rating), that means that the analysis was more based on examining suicidal ideation, not suicidal behavior. Despite these limitations, the analysis of this relatively large sample indicates, that there is no hint for an induction of suicidal behavior by rTMS treatment. In contrary our data suggest that during rTMS treatment suicidal risk decreases together with an improvement of other core symptoms of depression.

We call through our work for further prospective studies of rTMS in depression that focus more explicitly on the effect of rTMS on suicidal ideation and suicide risk. Moreover, our data suggest that further studies are warranted that directly investigate the effects of rTMS on suicidal ideation not only in patients with depression, but also in other psychiatric disorders.

## Conclusion

Based on the proposed data, there is no evidence that rTMS increases the risk for suicidality during the course of the treatment. Conversely, rTMS tends to reduce suicidal ideation. Our findings call for further rTMS controlled studies using large sample sizes and specific suicidality assessment measures to obtain more conclusive results.

## Data Availability Statement

The datasets for this manuscript are not publicly available because the approval from the ethic committee of university of Regensburg is restricted to publication of the data results - not the datasets itself - in the corresponding journals. Requests to access the datasets should be directed to: PD Dr. Martin Schecklmann, Email: martin.schecklmann@medbo.de.

## Ethics Statement

The studies involving human participants were reviewed and approved by Ethics committee of the University of Regensburg. Written informed consent for participation was not required for this study in accordance with the national legislation and the institutional requirements.

## Author Contributions

MA, PK, and TP recorded the data. MA, MD, and AM entered it in a database. MS performed the statistical analysis. MA, BL, and MS drafted the manuscript. All the authors designed the study, interpreted the data, and approved the final version of the manuscript.

## Conflict of Interest

The authors declare that the research was conducted in the absence of any commercial or financial relationships that could be construed as a potential conflict of interest.
